# Multi-Gene Testing Overview with a Clinical Perspective in Metastatic Triple-Negative Breast Cancer

**DOI:** 10.3390/ijms22137154

**Published:** 2021-07-01

**Authors:** Martina Dameri, Lorenzo Ferrando, Gabriella Cirmena, Claudio Vernieri, Giancarlo Pruneri, Alberto Ballestrero, Gabriele Zoppoli

**Affiliations:** 1 Department of Internal Medicine, University of Genoa, 16132 Genoa, Italy; martina.dameri@hsanmartino.it (M.D.); lorenzo.ferrando@edu.unige.it (L.F.); gabriella.cirmena@unige.it (G.C.); aballestrero@unige.it (A.B.); 2 Medical Oncology Department, Fondazione IRCCS Istituto Nazionale dei Tumori, 20133 Milan, Italy; claudio.vernieri@istitutotumori.mi.it; 3 IFOM, The FIRC Institute of Molecular Oncology, 20139 Milan, Italy; 4 Department of Pathology, Fondazione IRCCS Istituto Nazionale dei Tumori, 20133 Milan, Italy; giancarlo.pruneri@istitutotumori.mi.it; 5 School of Medicine, University of Milan, 20122 Milan, Italy; 6 IRCCS Ospedale Policlinico San Martino, 16132 Genoa, Italy

**Keywords:** next-generation sequencing, multi-gene testing, gene panels, breast cancer, metastatic triple-negative breast cancer, tumor mutational burden

## Abstract

Next-generation sequencing (NGS) is the technology of choice for the routine screening of tumor samples in clinical practice. In this setting, the targeted sequencing of a restricted number of clinically relevant genes represents the most practical option when looking for genetic variants associated with cancer, as well as for the choice of targeted treatments. In this review, we analyze available NGS platforms and clinical applications of multi-gene testing in breast cancer, with a focus on metastatic triple-negative breast cancer (mTNBC). We make an overview of the clinical utility of multi-gene testing in mTNBC, and then, as immunotherapy is emerging as a possible targeted therapy for mTNBC, we also briefly report on the results of the latest clinical trials involving immune checkpoint inhibitors (ICIs) and TNBC, where NGS could play a role for the potential predictive utility of homologous recombination repair deficiency (HRD) and tumor mutational burden (TMB).

## 1. Introduction

With the advent of massive parallel sequencing, the simultaneous evaluation of multiple genes has been greatly improved in terms of turnaround times, sequencing cost efficiency, and detection accuracy. Next-generation sequencing (NGS), in the forms of whole genome sequencing (WGS), whole exome sequencing (WES), or multi-gene testing, has been rapidly adopted in clinical practice. Gene panels, in combination with suitable bioinformatic pipelines, allow for the detection of germline or somatic mutations, copy number variations, genomic rearrangements, and tumor mutational burden in a single run starting from tissue biopsy—either fresh frozen or formalin-fixed paraffin-embedded (FFPE)—or, alternatively, from liquid biopsy (circulating tumor cells or circulating cell-free DNA/RNA). Hence, they represent a powerful tool for physicians to evaluate critical changes in genes related to cancer and to identify actionable molecular targets guiding the decision-making process. The analytical validity and clinical utility of gene panels have already been established for some cancer types [1,2]. In the field of breast cancer, international guidelines do not include multi-gene testing in patients’ care and management, but data suggest that they will be soon integrated [3,4]. Breast cancer is the cancer type with highest incidence worldwide [5], and despite the increased cure rate of this neoplasm and the increased life expectation of advanced disease, new efficient targeted therapies are required to improve breast cancer outcome. Triple-negative breast cancer, which is characterized by the lack of the expression of estrogen, progesterone, and HER2 receptors, is the most aggressive and deadly subtype. Despite the improvement of (neo)adjuvant therapies in recent years, which has led to a significant increase of cure rates in patients with limited-stage disease (stage I-III), metastatic TNBC (mTNBC) remains almost invariably incurable, with a median overall survival of 12–18 months [6,7]. Since cytotoxic chemotherapy remains the most effective but still largely unsatisfactory strategy in mTNBC treatment, new and effective therapeutic strategies are urgently needed. The possibility to comprehensively characterize human cancer through the NGS, and eventually other integrated approaches, opens the way not just to a deeper molecular understanding of cancer, and more specifically of TNBCs, but also (and above all) to a more appropriate and adequate classification of patients who may benefit from standard therapeutic approaches or experimental interventions in the context of clinical trials.

## 2. NGS Available Platforms for Multi-Gene Testing

Recently, NGS has been implemented in the clinical setting, especially in the field of oncology, for the identification of germline or somatic mutations, genomic rearrangements, and the global frequency of mutations in a neoplastic specimen, opening the way to personalized medicine [8]. This large-scale sequencing technology allows one to investigate the entire genome (whole genome sequencing, WGS), the exons within all known genes (whole exome sequencing, WES), or only exons of selected genes (targeted panels). Thus far, WGS is not commonly performed in clinical practice because it is rather expensive and time-consuming to carry out. In addition, it provides extremely complex results compared to WES, often without a clear transferability to clinical practice (e.g., information on non-coding regions) [9]. Nonetheless, the most modern WGS platforms such as Illumina NovaSeq 6000 are able to process a large amount of samples in relatively short turnaround times, making WGS more feasible. In contrast, WES can be generally considered the gold standard for genomic tumor characterization, as it provides the most complete information about the sample mutational landscape by analyzing the whole coding sequences of the genome with a deep average coverage (120–150×). However, WES also has significant limitations, including high costs, long turnaround times, a significant quantity of DNA input required for sequencing procedures, and large amounts of data to be analyzed and interpreted; together, these limitations make WES unsuitable for routine clinical use [10]. In contrast, the use of targeted gene panels covering only a subset of the genome focusing on a certain number of cancer-related genes is a more cost- and time-effective solution that goes beyond genome/exome-wide applications [11]. Targeted NGS panels are specifically designed to detect point mutations, small insertions and deletions, copy number variations (CNVs), fusion genes, or other structural aberrations using a small DNA input generally extracted from tissue biopsy or obtained through liquid biopsy [12]. Multi-gene testing is performed through NGS platforms, where genomic regions of interest are selected from the DNA sample before sequencing [13]. Two main target-enrichment methods occupy most of the NGS market, associated with different sequencing chemistries. The first method consists of an amplicon-based sequencing approach, which uses PCR and primers to generate multiple amplicons from the same initial sample. After library preparation, the amplicons, linked to their adapters, are conjugated with unique DNA barcodes in order to allow for sample multiplexing with subsequent cost-per-sample decreases on larger platforms [14]. The second target-enrichment method consists of the hybridization capture. Through this approach, DNA is fragmented by sonication or enzymatic digestion; then, fragments are hybridized with specific probes and ligated to adapters containing a sequence identifying the sample [14]. Both approaches have some advantages and disadvantages. The amplicon-based method, supported mainly by Agilent (HaloPlex and HaloPlex HS) and Thermo Fisher Scientific (Ion AmpliSeq), is usually valid for the detection of point mutations, small insertions, and deletions; it is faster, cheaper, and requires smaller DNA inputs compared to hybridization capture [15]. However, there are some limitations associated with the amplification strategy itself, including the risk of producing non-specific amplicons during the amplification step; in addition, amplicons are generally short (<10 kb), thus limiting the possibility to sequence large DNA regions in a single step [16]. On the other hand, the hybrid-capture based approach, typical of the Illumina platform, has the advantage of lower associated costs, and this allows one to cover larger regions of the genome (few megabases) and to detect not only point mutations but also chromosomal aberrations and rearrangements. Though the aforementioned approaches show a good concordance in sequencing depth, SNV calling, and indel calling, the hybridization capture-based method allows for higher sequencing uniformity when compared to the amplicon-based method [17]. Despite higher costs and the need for bigger amounts of input DNA and larger sequencers, hybridization capture is considered to perform better and to be more suitable when multi-gene testing NGS is applied to cancer research and diagnostics [14,18].

Multi-gene panels are mainly used in clinical practice for the detection of germline and somatic mutations in a wide range of tumors. Though there are some differences across different panels in terms of the number of genes included and genetic alterations evaluated, they all include well-known high-penetrance genes, as well as moderate- and low-penetrance genes and variants of unknown significance (VUS) [19]. The only two panels approved by FDA as companion diagnostic for solid tumors are FoundationOne CDx (324 genes) [20] and MSK-IMPACT (468 genes) [21], which are performed in CLIA (Clinical Laboratory Improvement Amendments)-certified laboratories. Nonetheless, there are many targeted panels that clinicians can order from certified facilities like Caris Life Science (592 genes) by Caris Molecular Intelligence, as well as commercially available panels to test in research laboratories like Thermo Fisher’s Oncomine Cancer Gene Mutation Panel v2 assay (143 genes), Oncomine Comprehensive Panel_v3 DNA (161 Genes), Oncomine Focus Assay DNA (52 Genes), Illumina’s TruSight Oncology 500 (523 genes), TruSight Tumor 170 (170 genes), and QIAGEN’s QIAseq Targeted DNA Panels just to name some of the most commonly advertised. Even though the majority of these panels include most of the commonly mutated cancer genes, in recent years, many institutions have decided to design their own targeted panels, including the most appropriate genes for their clinical or research purposes [22,23]. Hence, a large number of multiple-gene targeted panels are available today, and there is strong evidence that they may be used in a versatile manner, not only for the analysis of mutational hotspots but also to investigate fusion genes, CNVs, and tumor mutational burden (TMB) [24,25,26]. Table 1 shows a list of the most common multi-gene panel tests available on market, together with a summary of their main analytical features.

## 3. Multi-Gene Testing Clinical Applications for Breast Cancer

### 3.1. Gene Mutations

In breast cancer, approximately forty driver genomic alterations in well-known oncogenes and tumor suppressor genes (TSGs) such as *BRCA1/2*, *TP53*, *PIK3CA*, *ESR1*, *PTEN*, *PALB2*, *GATA3*, *KMT2C*, *NCOR1*, *AKT1*, *NF1*, *CDH1*, and *RB1*, as well as in a number of novel genes including *TBX3*, *RUNX1*, *CBFB*, *AFF2*, *PIK3R1*, *PTPN22*, *PTPRD*, *SF3B1*, and *CCND3*, have been reported [56,57,58]. Nonetheless, the present version (v93) of the Cancer Gene Census by COSMIC encompasses almost 500 cancer genes associated with breast cancer and in Tier 1, meaning that there is a high evidence of correlation between their mutations and cancer development [59]. In clinical practice, mutations in all of these genes are evaluated when WGS or WES are performed; conversely, when multi-gene testing is carried out, only a subset of these genes is evaluated, mainly based on their putative role in cancer development and progression. Almost every commercially available targeted panel contains crucial genes like *BRCA1/2*, *TP53*, *PIK3CA*, *ESR1*, and *PTEN*.

Currently, germline or somatic mutations in *BRCA1/2* genes are investigated in clinical practice for risk assessment, diagnosis, and treatment decision-making in breast cancer patients [60]. *BRCA* genes play a key role in the regulation of the cell cycle, cell proliferation, cell differentiation, and, importantly, in the process of DNA damage repair (DDR). As DDR pathways are intertwined signaling networks that recognize and repair mistakes arising during DNA replication and transcription, as well as during cell exposure to chemical and physical agents [61], their impairment results in homologous recombination repair deficiency (HRD), which is one of the main molecular mechanisms underlying cancer development and progression. Many works have demonstrated the correlation between each of the approximately 300 *BRCA1/2* mutations identified so far and HRD-mutated breast cancer onset, especially for the TNBC subtype [62,63], making it clear that at least *BRCA1/2* germline assessment should be performed in people with a family history of breast cancer. While *BRCA* testing was initially performed as a single-gene test and by a single company (BRACAnalysis CDx, Myriad Genetics), the US Supreme Court’s 2013 decision against gene patenting made it possible for many companies to develop new multi-gene cancer susceptibility tests including *BRCA1/2* genes [64,65], which are now commonly used in clinical practice. Of note, *BRCA1/2* germline mutational status is routinely evaluated for the selection of advanced breast cancer patients who may benefit from the poly-adenosine diphosphate–ribose polymerase (PARP) inhibitors olaparib and talazoparib [66]. More recently, the positive results of ad interim analysis of the OlympiA trial brought the prospective of *BRCA1/2* testing in the early setting for therapeutic purposes [67]. Apart from cancer predisposition tests, *BRCA* genes are also included in all of the other multi-gene panels for somatic mutation detection, even though they are not among the most frequent somatic mutated genes in breast cancer. In fact, somatic mutations are far more recurrent in *TP53*, *PIK3CA*, *ESR1*, and *PTEN*. *TP53* is the most commonly mutated gene in human cancers, and it can be also associated with hereditary cancer syndromes. In breast cancer, *TP53* mutations, typically missense or frameshift mutations that lead to a loss-of-function effect [68], are more frequent in estrogen receptor (ER)-negative breast cancer and are detected in up to 80% of all TNBC specimens [69]. Though *TP53* mutational status is evaluated by all gene panels, its clinical relevance is not completely supported by clinicians; in fact, there have been discordant studies that have shown how a *TP53* mutation can have a detrimental, neutral, or beneficial effect on clinical outcomes [70,71,72]. On the contrary, a promising therapeutic target and biomarker for breast cancer is represented by the phosphatidyl-inositol 3-kinase PI3K/AKT/mTOR pathway. *PIK3CA*, which encodes for the p110α subunit of PI3K, is the second most commonly altered gene in breast cancer and is mutated in 20–40% of ER-positive tumors, as well as in TNBC [73]. *PIK3CA* mutations within the helical and the kinase domains cause an hyperactivation of PI3K pathway, resulting in uncontrollable cell proliferation. Similar biological effects result from *PTEN* loss-of-function, as this tumor suppressor protein is a negative regulator of *PIK3CA*. *PTEN* alterations and *PIK3CA* hotspot mutations in exons 2, 5, 10, 14, 20, or 21 are easily detected through NGS and multi-gene testing [74]. In the preclinical and clinical settings, *PIK3CA* mutations are now more and more often considered a biomarker of tumor sensitivity/resistance to specific treatments, including paclitaxel, trastuzumab, and endocrine treatment [75]. Despite controversial evidence regarding the prognostic value of *PIK3CA* alterations in TNBC, clinical trials are evaluating the efficacy of PI3K, AKT, and mTOR/PI3K inhibitors, alone or in combination with other therapies like androgen receptor inhibitors, in TNBC patients [76]. Another clinically relevant and frequently mutated gene in breast cancer is *ESR1*. Notably, nonsynonymous *ESR1* mutations affecting the ligand-binding domain of ER generally cause a constitutive activation of ER, which results in enhanced cell proliferation and resistance to endocrine therapy in ER-positive breast cancer [77,78]. Mutation analysis through digital droplet PCR (ddPCR) and NGS, including multi-gene testing, revealed that *ESR1* mutations are predominant in the metastatic setting rather than in primary tumors and paved the way for the evaluation of *ESR1* mutational status for therapy decision-making in ER-positive breast cancer [79].

### 3.2. Gene Amplifications/Deletions

CNVs represent one of the most prevalent genomic alterations in breast cancer, thus making the detection of clinically relevant CNVs of prognostic and therapeutic value in clinical practice [80]. Certain CNVs, such as the amplification of *ERBB2* gene, occur at a high frequency in specific breast cancer phenotypes, such as HER2-positive breast cancer (20–40% of the cases). Notably, *ERBB2* amplification causes the overexpression of tyrosine kinase receptor HER2, which triggers the downstream hyperactivation of PI3K/AKT and mitogen-activated protein kinase (MAPK) pathways and which results in tumor cells proliferation and survival. The amplification of the *ERBB2* gene, which is located on the chromosome region 17q12, is already an established therapeutic target for the selection of patients benefiting from the use of anti-HER2 monoclonal antibodies or tyrosine kinase inhibitors [81]. While previous studies have reported on germline CNVs (21), current research is mainly focused on somatic CNVs as targetable genetic alterations. Common methods for CNV detection are PCR, fluorescence in-situ hybridization (FISH), comparative genomic hybridization (CGH), and whole genome single-nucleotide polymorphism (SNP) arrays. Though fast and relatively cheap, in recent years, these approaches have been replaced by NGS technology, which includes CNVs analysis when either WGS/WES or multi-gene testing are performed. In fact, many commercially available targeted panels (such as MSK-IMPACT and Oncomine) and in-house developed panels are suitable for the detection of gene amplifications and deletions [80,82,83]. Comparative studies have been carried out to show the overall concordance between the amplification calls performed through NGS panels or through the current standard of assessment being FISH [84,85]. Besides *ERBB2*, other potentially actionable genes that have been found to be often amplified in breast cancer, including TNBC, are *NOTCH1/2/3*, *MYC*, *FGFR1/2*, and *EGFR*, with *EGFR* amplification associated with poor patients outcome [86,87]. With regard to deletions, they are less frequent in breast cancer and might be less clinically relevant when compared with somatic mutations and amplifications. However, the most common deletions found in breast tumors involve *PTEN* (typically of TNBC), *CDH1*, *CDKN2A/2B*, *RUNX1/CBFB*, *RB1*, and *INPP4B* [56].

### 3.3. Genomic Rearrangements

Genomic rearrangements resulting from balanced or unbalanced translocations usually lead to the formation of fusion genes and proteins. Though fusion genes have been primarily investigated in hematological malignancies, notably leukemias and lymphomas (with the best-known example of *BCR-ABL1* fusion protein in chronic myeloid leukemia) they have also been found in solid tumors, such as the case of the *EML4-ALK* fusion in lung adenocarcinoma [88]. Therefore, it is becoming clearer and clearer that fusion genes can play an important role in cancer development and progression, and they may be clinically actionable genomic alterations with potential therapeutic impact. In clinical practice, the presence of fusion genes is assessed through FISH or immunohistochemistry (IHC) techniques, which might not be sufficiently reliable for multiplex analysis and more comprehensive tumor profiling [89]. NGS, in the form of WGS or RNA sequencing, has been rapidly implemented for detecting known fusion genes as well as for finding new ones [90]. Though RNA-seq appears to be the most popular approach [91,92], data suggest that parallel sequencing assays, especially Illumina TruSight Tumor 170, TruSight Oncology 500, and QIAGEN QIAseq RNAscan Custom Panel, are feasible for the identification of fusion genes [62]. Many fusion genes have been discovered in breast cancer. Some large genomic rearrangements involve *BRCA1/2* and can be reliably detected through an in-house developed test, the BRACAnalysis Rearrangement Test (BART), which is a quantitative multiplex endpoint PCR assay followed by Sanger sequencing [93]. Other recurrent rearrangements in breast cancer are fusions including *MAGI3-AKT3*, *FGFR3-TACC3*, *BCL2L14–ETV6*, and *ESR1–CCDC170* fusions. *MAGI3-AKT3* fusion results in the constitutive activation of AKT kinase, and it is now considered a possible target for ATP-competitive AKT small-molecule inhibitors [94]. However, the apparent low frequency of this alteration in TNBC has reduced the interest in conducting clinical trials with AKT inhibitors [95]. A more promising gene fusion in TNBC involves the FGFR3 kinase domain and the upstream region of the coiled-coil domain of transforming acidic coiled-coil 3 (TACC3) protein. This fusion, which promotes cell proliferation and cancer formation in vivo, could be a targetable driving alteration predicting tumor response to FGFR inhibitors [96,97]. Recently, the *BCL2L14–ETV6* fusion has been exclusively detected in TNBC specimens, especially in neoplasms with characteristics of high clinical aggressiveness and associated with poorer prognosis. Though this rearrangement has been primarily studied in in vitro cultured TNBC cell lines, it seems to be correlated with resistance to paclitaxel; however, further preclinical and clinical studies are required to confirm this association [98]. Another common fusion that is found in ER-positive breast cancer is *ESR1–CCDC170* fusion (6–8% of luminal B breast tumors). No specific targeted therapies exist for this genomic alteration, and elucidative studies are expected to shed light on the various infrequent and more recurrent *ESR1* fusions, as well as their role in endocrine resistance of primary and metastatic ER-positive breast cancer [90]. Finally, there is one fusion gene subtype, namely *NTRK* fusions, which has been considered as an actionable genomic target in different cancer types and for which a targeted therapy is under investigation [91]. However, its frequency in breast cancer is very low.

### 3.4. Overexpression and Downregulation

Gene expression has been rapidly introduced into clinical cancer management. mRNA expression profiles are considered useful clinical tools for therapeutic stratification of cancer patients [99]. There are many RNA-based, commercially available assays in the form of medical devices or tests with a prognostic value for breast cancer (OncotypeDX, MammaPrint, Prosigna, EndoPredict, Breast Cancer Index, Mammostrat) [100]. The clinical utility of such gene expression assays has been investigated in dedicated clinical trials [101,102,103], with preliminary data strongly confirming that they are going to have an impact on clinical practice.

In the context of TNBC, gene expression immune profiles could be even more informative to predict the presence of tumor-infiltrating specific immune cell populations. For instance, the Lehman classification of human TNBCs led to the identification of six different subgroups, which are characterized by different genomic profiles, clinical behaviors, and responses to standard antitumor therapies [104]. One of the Lehman TNBC subtypes is the immunomodulatory subtype, which is characterized by the expression of many genes related to antigen presentation, intratumor immune cell infiltrate, and cytokine signaling (e.g., TNF and NFkB) [104]. However, the Lehman classification is limited by the fact that it is affected by the presence of stromal cells in tumor specimens, such as lymphocytes and mesenchymal cells, thus making it unstable. The acknowledgment of this limitation led to a new classification of TNBC into only four subsets, with the disappearance of the IM subtype [105,106]. Another classification of TNBC, on the basis of gene expression profiles, led to the identification of four subtypes, namely those of luminal androgen receptor (LAR), mesenchymal (MES), basal-like immune suppressed (BLIS), and basal-like immune activated (BLIA). The LAR subtype is characterized by the overexpression of androgen receptor and by an upregulation of the estrogen signaling pathway [107]. MES, being the mesenchymal subtype, expresses low levels of genes associated with cell division and proliferation, as well as high levels of *HOX* genes and genes related to stemness such as *BCL2* and *BPM2* [108]. BLIS is the most proliferative subtype, and it is also associated with worse patient outcomes compared to the other subtypes; nonetheless, it has shown an increased expression of genes related to proliferation (*CENPF* and *BUB1*) and a downregulation of genes implicated in the immune response [108]. Lastly, the BLIA subtype is characterized by *CTLA4* overexpression, enhanced interferon and IL-2 signaling, JAK/STAT pathway and NFkB activation, and better disease-free survival [109].

Similarly to immune profiles, gene expression immune signatures, which evaluate the expression of dozens of genes associated with different immune pathways and immune cells, could provide more reliable predictive information. In the context of cytotoxic chemotherapy, an enrichment of specific gene expression immune signatures containing crucial effectors of antitumor immune response, such as granzymes and perforin, have been associated with a better prognosis in patients with aggressive BC subtypes, including TNBC [110,111].

Besides immune signatures, several other genes have been found to be differentially expressed between breast cancer subtypes, more specifically in TNBC. Integrating genomics analyses have identified subsets of genes that are typically overexpressed in TNBC compared to non-TNBC breast cancers. Such genes, which are only in part known and associated with specific biological functions, are generally involved in crucial processes like cell growth, apoptosis, angiogenesis, and response to drugs and estradiol, as well as contributing to cancer progression [112]. For instance, *MTBP* (Mdm2 Two-binding protein), a positive regulator of the Myc gene (which regulates cell-cycle progression and proliferation) was reported to be dysregulated in TNBC. The mRNA overexpression of this gene has been associated with decreased TNBC patients’ survival [113]. Similarly, high mRNA levels of the *ETV4* gene (ETS translocation variant 4), belonging to the family of ETS transcription factors, have been related to metastasis dissemination and poor patients outcome in TNBC [114]. Another interesting gene that has been found to be overexpressed in TNBC is *COX2*, encoding for cyclooxygenase 2—one of the main inflammatory genes that regulates the production of prostaglandins. In vitro and in vivo studies have shown that high mRNA and protein levels of *COX2* play a role in tumor progression and are biomarkers of high tumor grade and poor prognosis for breast cancer patients [115]. Nonetheless, further studies have investigated and confirmed the correlation between high *COX2* levels and the expression of tumor-promoting genes such *EGR2* and *IL6*. Interestingly, *COX2* expression has been correlated with aromatase *CYP19A* expression in ER-, PR-, and Her2-positive breast cancers [116]. Similarly to *COX2*, the *ALOX5* gene, encoding for arachidonate 5-lipoxygenase, has been associated with a high expression of further tumor-promoting genes involved in mutagenesis and immunosuppression [116].

Though many studies have focused on mRNA expression profiles in breast cancer, there have been a relatively limited number of clinical trials involving TNBC that have evaluated gene expression alone with the aim of driving therapy decision-making. Tumor transcriptomics could be of help in order to allow for a better understanding of cancer biology and actionable targets; however, it should be integrated with WGS and WES analyses [117].

### 3.5. Genomic Signatures

Genomic signatures represent the effect of mutational processes on the cancer genome due to the exposure to exogenous or endogenous mutagens. Single-base or doublet-base substitutions, small insertions, deletions, genomic rearrangements, and chromosome CNVs are all examples of mutational patterns that may hit genes involved in the mechanisms of DNA damage response, DNA repair, and DNA replication [118]. In breast cancer, the most common signatures are signature 1B, 2, 3, 8, and 13 [119]. In particular, signature 3 and 8 have been observed to be strongly associated with *BRCA1/2* somatic or germline mutations or promoter methylation, which are quite common features in TNBC [120]. Homologous recombination (HR) is one of the main mechanisms of DNA repair after damage-caused double-strand breaks (DSBs), and mutations in the genes involved in this process such as *BRCA1*, *BRCA2*, *RAD51*, and *PALB2* impair HR, thus causing HRD. HRD tumors, due to the inefficient DNA reparation they perform, are more sensitive to DNA-damaging agents such as cisplatin, taxanes, and anthracyclines, as well as to PARP inhibitors [121]. As HRD has been studied as a possible biomarker for the response to these therapies, several assays have been developed to measure it using array-based comparative genomic hybridization (aCGH), SNP, analysis and NGS [122]. Two assays based on SNP analysis and FDA-approved as companion diagnostic are mainly used in the clinical setting: the MyChoice HRD test by Myriad Genetics, which combines three different genomic alterations being loss of heterozygosity (LOH), telomeric allelic imbalance (TAI), and large-scale transition (LST) and which provides a comprehensive HRD score [123], and the FoundationFocus CDx BRCA LOH that detects *BRCA1/2* mutations and the total amount of LOH in the genome [124]. However, in recent years, there has been a migration from SNP arrays to NGS techniques for the assessment of the HRD score. It was demonstrated that there is no significant difference between SNP array-based and NGS-based calculation of the HRD score [125]. In this context, new methods and models have been developed using WES or WGS. A WGS-based new model that accurately identifies *BRCA1/2* mutations in HRD tumors is HRDetect. This model is considered a new specific predictor of HRD for breast cancer and is more efficient than WES in distinguishing *BRCA1*- rather than *BRCA2*-deficient tumors [126]. Furthermore, in order to detect HRD in all the different cancer types on the basis of mutation profiles, a random forest-based Classifier of Homologous Recombination Deficiency (CHORD) was used in tandem with WGS. Through CHORD, it has been possible to distinguish between a *BCRA1*-type deficiency, typical of breast and ovarian cancer, and a *BCRA2*-type deficiency, which comprises mutations in *BRCA2*, *RAD51*, and *PALB2*, typical of prostate and pancreatic cancer [127]. In addition, this study demonstrated that HRD is higher in the metastatic rather than in the primary tumor [127]. Finally, another way of evaluating HRD using NGS technology is through multi-gene panels. The majority of commercially available gene panels used for mutation detection contains genes for HRD, so it is relatively easy to investigate their mutational status [128].

Another valuable genomic signature is TMB, which is defined as the number of somatic non-synonymous mutations per megabase (mut/mb) in a neoplastic specimen [119]. TMB is already recognized as a good biomarker of immune checkpoint inhibitor (ICI) response, independently of PD-L1 expression, in NSCLC, melanoma, and bladder cancer [129,130]. This is because tumors with high values of TMB are more likely to express new immunogenic antigens; this, in combination with ICIs, can result in the alteration of the tumor microenvironment and, consequently, in the activation of the immune system against cancer cells [131]. Though breast cancer is not particularly immunogenic compared to other malignancies [132], it has been demonstrated that TMB can also be a predictive biomarker for ICI treatment and prognosis in this kind of tumor, more specifically in TNBC because this is a subtype with high mutation rates [133,134]. TMB is generally calculated through WES, but, as this kind of analysis is rather time-consuming and expensive, it is more frequently estimated using gene panels [10]. In many cases, the NGS-targeted panels currently used for the oncogene sequencing and detection of actionable mutations include TMB testing. Nonetheless, TMB assessment is often performed through specific and dedicated gene panels like Thermo Fisher’s Oncomine Tumor Mutation Load Assay, SureSelect XT HS custom TMB and human all Exon v6 panel, QIAseq TMB panel, and NEOplus v2 RUO panel [135]. These panels have been analyzed through empirical and in silico approaches, and it has been found that they provide a reliable approximation of TMB in accordance with TMB values calculated with WES [41]. Other gene panels used for research application and suitable for proper TMB determination are Tempus xT, NeoTYPE Discovery Profile, CANCERPLEX, Foundation Medicine bTMB assay, Guardant360, and PlasmaSELECT64. In addition, some institutions and companies have developed their own targeted panels for TMB calculation that have obtained analytical validity in many cases [31,136]. In general, data suggest that targeted panel size should be at least 1.1 Mb in order to accurately estimate TMB [137]. Smaller panels were shown to overestimate TMB, especially in those cancer types characterized by low or intermediate levels of TMB. Even though the question is still debated, many studies have demonstrated that accuracy drops when gene panels are smaller than 0.5 Mb, losing correlation between panel-based TMB and WES TMB values [137]. Moreover, another aspect to be kept in mind is the issue of TMB calculation on FFPE-degraded samples. In fact, TMB estimation could be altered due to an artefactual increase in deaminations [12]. Gene panels that are used to determine TMB, especially in the clinical/diagnostic setting, often contain genes like *CD274* and *PDCD1* that encode for PD-L1 and PD1, respectively, as well as genes for HRD. High values of TMB were found in association with mutations in such genes in TNBC [138], suggesting that this type of tumor, due to its high mutation rate, could benefit from ICIs and patients undergoing therapy could be selected and monitored to evaluate TMB. However, although TMB is emerging as an interesting biomarker for the stratification of ICI response, standardization in its assessment is still lacking. There is an unmet need for harmonization and normalization across platforms and systems for what concerns pre-analytical, methodological, and analytical factors, including cut-off values with the aim to increase TMB consistency and reliability, as well as to implement its evaluation in the clinic [139].

## 4. Clinical Utility of Multi-Gene Testing for Metastatic Triple-Negative Breast Cancer

Several clinical trials have indicated that using multi-gene testing to personalize cancer therapies can have a positive impact on tumor response rates and patient progression-free survival, as well as on finding new molecularly-targeted treatment indications [140,141,142]. Multi-gene testing is considered the most appropriate option for patients with advanced or metastatic cancers with limited standard-of-care options in order to assess several actionable genomic alterations at the same time [143]. In addition, this type of testing can be of help for selecting patients who may be included in clinical trials [144,145]. Together, the available clinical evidence indicates that tumor multi-gene testing could provide useful biological information to characterize TNBC specimens, even though the clinical utility of NGS multi-gene panels for breast cancer care and management is still debated and their use is not fully recommended in routine clinical practice [146,147,148]. Because mTNBC lacks the expression of estrogen, progesterone, and HER2 receptors and therapies against these targets are ineffectual, gene panels could help in investigating the comprehensive mutational status, including the presence of targetable mutations in genes commonly altered in mTNBC such as *BRCA1/2*, *TP53*, and *PIK3CA. BRCA1/2* genes are included in all of the commercially available gene panels for hereditary breast and ovarian cancer risk assessment and management because they are well-known cancer susceptibility genes. The clinical utility of such panels, which also include other susceptibility genes like *PALB2*, *PTEN*, *STK11*, *TP53*, *ATM*, *BARD1*, *BRIP1*, *CDH1*, and *CHEK2*, is widely recognized [149,150], and many studies have supported the idea that multi-gene testing should be offered to all women diagnosed with breast cancer and not only to those who have a family history of breast cancer or who respect specific clinical criteria [151]. In addition, *BRCA1/2* mutations predict the clinical efficacy of PARP inhibitors olaparib and talazoparib, which are FDA-approved for patients with germline *BRCA1/2* mutations in the advanced breast cancer setting, including mTNBC [66,152]. Regarding genomic alterations other than *BRCA1/2* mutations, there are many ongoing clinical trials aimed at targeting alterations in *TP53* and *PIK3CA* genes. However, the majority of clinical trials conducted so far have shown no evident benefit of using gene testing for treatment decision-making compared to treatment at physician’s choice [153]. Thus far, the most promising application of multi-gene testing for mTNBC consists of the identification of mutational signatures, notably HRD and TMB. In the context of mTNBC, which is the breast cancer subtype characterized by the highest mutation rate, HRD is detected in a consistent percentage of cases and the HRD score is often elevated [121]. The HRD score has been used in different clinical trials involving mTNBC patients to predict the response to treatments including standard neoadjuvant chemotherapy [123], carboplatin added to anthracycline/taxane-based neoadjuvant chemotherapy [154], and PARP inhibitors [155]. It has also been suggested that HRD could have a predictive value in terms of clinical benefit from ICIs. The rationale behind a potentially higher clinical benefit of ICIs in patients with tumors showing HRD is the fact that HRD results in increased genomic instability, an enhanced neoantigen formation, higher TMB, and higher anti-tumor immune response in mTNBC [156]. However, the correlation between HRD and ICI response is still to be demonstrated. Instead, TMB is currently considered the most promising biomarker of response to immunotherapy in mTNBC. This is because the high values of TMB subtend neoantigen formation on cancer cell surface and the activation of the immune system. Therefore, gene panels reliably estimating TMB scores could have an important clinical utility in selecting mTNBC patients who may benefit from immunotherapy [157]. This can be confirmed by a number of clinical trials that involved cancer types such as NSCLC, bladder cancer, and melanoma but not mTNBC [158]. Regarding the other above-mentioned genomic alterations that can be potentially evaluated through multi-gene testing, namely gene amplifications or deletions and fusion genes, only a limited number are under investigation in the mTNBC setting (e.g., *PIK3CA* mutations in combination with androgen receptor expression and *NTRK* fusions), but none of them have actually been investigated in clinical trials using targeted panels. Taken together, such evidence suggest that further clinical trials involving mTNBC are needed with the aim of investigating suitable biomarkers to predict the response to targeted therapies. With regard to immunotherapy, a combination TMB and HRD assessment may be of help in order to select patients who may benefit from this kind of therapy.

## 5. The Dilemma of Immunotherapy in Metastatic Triple-Negative Breast Cancer

Among different breast cancer subtypes, TNBC is characterized by a higher number of tumor-infiltrating T lymphocytes (TILs). An increased number of TILs at diagnosis, as evaluated by hematoxylin-eosin staining, is associated with higher rates of pathologic complete responses (pCRs) after neoadjuvant chemotherapy following surgery in limited-stage TNBC patients [159], as well as with better patient survival after surgery that is either preceded or followed by chemotherapy [159,160]. In addition, as previously mentioned, TNBC is characterized by a high TMB, with 1.68 mutations per megabase (Mb) [57]. Notably, a high TMB leads to higher response rates during single-agent immunotherapy in human cancers. Together, these data led to the hypothesis that TNBC is more immunogenic when compared to other breast cancer subtypes and, therefore, that it is potentially more likely to respond to ICIs. Recently, the phase III IMpassion031 trial showed that adding atezolizumab to preoperative nab–paclitaxel followed by anthracycline-cyclophosphamide chemotherapy resulted in a significant increase of pCR rates (from 41% to 58%), with PD-L1-positive tumors being associated with significantly higher pCR rates in both the control and experimental arms (49% vs. 69%, respectively) [161]. On the other hand, in the phase III NeoTRIP trial, adding atezolizumab to preoperative carboplatin-paclitaxel chemotherapy did not result in a statistically significant increase of pCR rates, while intratumor PD-L1 expression was associated with significantly higher pCR rates in both the experimental and control groups [162]. Though the efficacy of ICIs in combination with preoperative chemotherapy has not been uniformly demonstrated in the phase III trials conducted so far, the available evidence supports the utility of adding ICIs to standard-of-care preoperative chemotherapy in TNBC patient subsets, thus providing proof-of-concept demonstration of the efficacy of immunotherapy.

In the context of mTNBC, the utility of ICIs is much more uncertain, likely because advanced cancers are characterized by the accumulation of immunological dysfunctions that finally limit the efficacy of drugs targeting single aspects of tumor-induced immune suppression. In recent years, phase I/II clinical trials have revealed that single-agent anti-PD1/PD-L1 monoclonal antibodies (mAbs) have antitumor activity in subsets of mTNBC patients, especially those with PD-L1-positive disease [163,164]. Based on these data, as well as on the synergistic antitumor activity of immunotherapy and chemotherapy in other advanced neoplasms (such as non-small cell lung cancer [165,166]) and in patients with limited-stage TNBC [161,167], two randomized, phase III trials investigated whether adding the anti-PD-L1 monoclonal antibody atezolizumab to first-line chemotherapy is able to improve clinical outcomes in mTNBC patients. The first of these two trials, the IMpassion130 study, assessed the efficacy of combining atezolizumab with first-line nab–paclitaxel in improving patient PFS and OS. The two end-points were evaluated both in the intention-to-treat (ITT) population and in the subset of patients with PD-L1-positive neoplasms, as defined by PD-L1 immunohistochemistry expression in ≥1% of cancer cells by a VENTANA PD-L1 SP142 assay. Atezolizumab was associated with a statistically significant improvement of patients PFS and OS in both the ITT population and in the subset of patients with PD-L1-positive disease but not in patients with PD-L1-negative tumors [7,168]. Based on these results, the nab–paclitaxel–atezolizumab combination was approved by the US Food and Drug Administration and by the European Commission as a first-line therapy in patients with PD-L1-positive mTNBC. However, since nab–paclitaxel is not widely approved in European countries as a first-line therapy for mTNBC, the European trial Impassion131 was subsequently initiated to investigate whether adding atezolizumab to standard-of-care, first-line paclitaxel would result in improved PFS and OS in mTNBC patients. The population of patients enrolled in the IMpassion131 trial was similar to that enrolled in the IMpassion130 study. The primary results of this study were reported at the ESMO 2020 Congress: quite disappointingly, adding atezolizumab to paclitaxel did not improve patients’ PFS or OS in either the PD-L1 cohort or the ITT population [169]. The observed discrepancies in the results of the Impassion130 and Impassion131 trials could have resulted from several factors, including: (1) the fact that paclitaxel, but not nab–paclitaxel, requires premedication with steroids, which might reduce atezolizumab efficacy; (2) the different impacts of paclitaxel and nab–paclitaxel on tumor-infiltrating macrophages and lymphocytes, with nab–paclitaxel potentially displaying more potent stimulatory activity on T lymphocytes; and (3) the lack of a reliable biomarker predictive of benefit from atezolizumab. Regarding the last point, although PD-L1 immunohistochemistry evaluation through the VENTANA SP142 assay is simple and well-reproducible, it might not be exhaustive in discriminating patients more or less likely to benefit from ICIs. The main limitation of PD-L1 assessment by IHC analysis is that it only reflects one single aspect of intratumor immune suppression, i.e., the binding of PD-L1 in cancer cells and/or macrophages to PD1 on tumor-infiltrating T lymphocytes, which results in immunosuppressive effects. Though the PD1/PD-L1 interaction is one crucial aspect of tumor-mediated immune suppression, as well as the target of anti-PD1/PD-L1 monoclonal antibodies, it might not fully reflect the complexity of tumor immune contexture and, in particular, the presence of different tumor-infiltrating immune cell populations [163]. For this reason, more complex biological evaluations, such as the assessment of genomic or gene expression tumor immune profiles or the evaluation of TMB, could provide valuable information that more reliably reflects the complexity of tumor immune contexture. However, the search for these biomarkers should occur in the context of investigational studies, since no established genomic or transcriptomic predictors of clinical benefit from immunotherapy have been identified so far.

In parallel, since the use of PARPi in advanced BC patients bearing germline inactivating alterations in *BRCA1* or *BRCA2* genes has resulted in improved patient PFS [66,152] and, in the case of olaparib in patients not previously treated with chemotherapy, in improved OS [170], the assessment of germline *BRCA1/2* status (where indicated) should parallel the intratumor evaluation of PD-L1 by IHC for the choice of the best therapy for patients with advanced mTNBC. Based on currently available data, patients with PD-L1-positive mTNBC are candidates to receive first-line nab–paclitaxel–atezolizumab, regardless of germline *BRCA1/2* status; patients with germline mutations in *BRCA1/2* genes and PD-L1-negative neoplasms by IHC are candidates to receive first-line olaparib/talazoparib; finally, patients with PD-L1-negative tumors and wild-type *BRCA1/2* status are candidates to receive standard chemotherapy (either single-agent or combination chemotherapy).

## 6. Methods

The following key strings were used for searching articles to be reviewed for the present manuscript in the PubMed, Google Scholar and Scopus research browsers: (([“breast cancer” OR “triple-negative breast cancer” OR “metastatic triple-negative breast cancer”] AND [genetics OR “next-generation sequencing” OR “multi-gene testing” OR “genomic alterations”])), ((“breast cancer” OR “triple-negative breast cancer” OR “metastatic triple-negative breast cancer”] AND [immunotherapy OR “immune checkpoint inhibitors”] AND [“clinical trials” OR “clinical practice”])). Only full articles in the English language published in the last ten years were considered for further review. References contained in the selected articles and not identified in our first research were also searched for, and the related articles were included in our review if suitable. The most recent articles with close relevance to metastatic TNBC and multi-gene testing were prioritized. 2010 was established as the starting year of the search in order to focus our review on the latest advances concerning the topic, although references of interest contained within those articles were screened as well. Great importance was also given to “Clinical study” and “Review” articles dealing with the topic. Among the remaining articles, the ones relating to breast cancer, genomic alterations, and multi-gene testing were considered first.

## 7. Conclusions

Multi-gene testing is being increasingly adopted in clinical practice and is going to change it deeply. However, the use of gene panels today is not homogeneous and is prevalent among certain cancer types that do not include mTNBC. Multi-gene testing should be largely implemented in the clinical setting, not only for a better understanding of tumor biology but also to drive cancer care and management and start new clinical trials with the aim of identifying novel targeted therapies. With regard to mTNBC, multi-gene testing should be considered and accepted as a valuable approach to select patients who may benefit from the restricted number of available target-specific therapies.

## Figures and Tables

**Table 1:** A comprehensive list of the most common commercially available multi-gene panels and multi-gene tests.

Test name	Company/Institution	No. of Genes	Coverage (Mb)	TMB Assessment	Other Applications	Sequencing Platform	Sample Type	Turnaround Time	Reference
SureSelect XT HS custom TMB and human all Exon v6 panel	Agilent Technologies	361	3.1	Yes	SNVs and indels	Illumina HiSeq, NextSeq and NovaSeq	FFPE	2–3 days	[27]
Variant Plex solid tumor	Archer	67	0.051	No	SNVs, indels, and CNVs	Illumina NGS systems	Fresh frozen tissue and FFPE	1 day	[28]
Reveal ctDNA 28	Archer	28	nd	No	Variant detection, and CNVs	Illumina NGS systems	ctDNA, solid tumors	nd	[29]
Caris Molecular Intelligence TumorSeek	Caris Life Sciences	592	1.4	Yes	SNV, indels, CNVs, and MSI	Illumina NextSeq 500	FFPE	14 days	[30]
OncoPanel	Dana Farber Cancer Institute	282	1.4	No	SNVs, indels, CNVs, and structural variants	Illumina HiSeq2500	Fresh frozen tissue and FFPE	nd	[31]
FoundationOne CDx	Foundation Medicine	324	0.8	Yes	SNVs, indels, structural rearrangements, CNVs, MSI, and HRD status	Illumina HiSeq 4000	FFPE	10 days or less	[20]
FoundationOne Liquid CDx	Foundation Medicine	324	0.8	Yes	SNVs, indels, structural rearrangements, and CNVs	Illumina NovaSeq 6000	Peripheral whole blood	10 days or less	[32]
Guardant360	Guardant Health	74	nd	No	SNVs, indels, fusions, amplifications, and MSI	Illumina HiSeq 2500	Plasma	7 days	[33]
GuardantOMNI	Guardant Health	500	2.1	Yes	SNVs, indels, CNVs, and fusions	Guardant digital sequencing platform	Plasma	nd	[26]
TruSight Oncology 500	Illumina	523	1.94	Yes	SNVs, indels, structural rearrangements, CNVs, and MSI	Illumina NextSeq 500–550 systems	FFPE	4–5 days	[25]
TruSight Oncology 500 ctDNA	Illumina	523	1.94	Yes	SNVs, indels, structural rearrangements, CNVs, and MSI	Illumina NovaSeq 6000	Peripheral whole blood	5 days	[34]
TruSight Tumor 170	Illumina	170	0.53	No	SNVs, indels, somatic and structural variants, and CNVs	Illumina NGS systems	FFPE, low-input samples	4–5 days	[35]
TruSight Tumor 15	Illumina	15	0.044	No	Indels and somatic variants	Illumina MiniSeq, MiSeq	FFPE	36 h	[36]
TruSight Cancer	Illumina	94	0.255	No	Germline variants	Illumina MiniSeq, MiSeq, NovaSeq 550	FFPE	3 days	[37]
CANCERPLEX	Kew Inc	435	2.8	Yes	SNVs, indels, CNVs, traslocations, and MSI	Illumina NGS systems	FFPE	7–10 days	[38]
MSK-IMPACT	MSKCC	468	1.5	Yes	Somatic mutations, structural variants, CNVs, and MSI	Illumina HiSeq 2500	FFPE	19 days (median)	[21]
MSK ACCESS	MSKCC	129	0.4	No	SNVs, indels, CNVs, and structural variants	Illumina HiSeq 2500 or NovaSeq 6000	Peripheral whole blood and other body fluids	16 days	[39]
NeoTYPE Discovery Profile	NEO New Oncology	323	nd	Yes	SNVs, indels, CNVs, fusions, and MSI	Illumina NGS systems	FFPE	14–17	[40]
NEOplus v2 RUO panel	NEO New Oncology	>340	1.1	Yes	SNVs, indels, CNVs, and MSI	Illumina NGS systems	FFPE	nd	[41]
PlasmaSELECT64	PGDx	54	0.78	No	SNVs, indels, and MSI	Illumina NGS systems	Plasma	14–21 days	[42]
PGDx elio plasma complete	PGDx	521	nd	Yes	SNVs, indels, CNVs, traslocations, MSI, and LOH	Illumina NextSeq 550Dx	Plasma	7–8 days	[43]
PGDx elio tissue complete	PGDx	521	nd	Yes	SNVs, indels, CNVs, traslocations, MSI, and LOH	Illumina NextSeq 550Dx	FFPE	7–8 days	[44]
QIAseq Targeted DNA Panels	Qiagen	<100	nd	No	SNVs, short indels, and CNVs	Illumina NGS systems or Ion Torrent NGS systems	FFPE, plasma/serum, fresh or frozen tissue, cell lines	nd	[45]
GeneRead DNAseq Targeted Panels V2	Qiagen	160	0.7	No	SNVs, indels, CNVs, and fusions	Illumina NGS systems or Ion Torrent NGS systems	FFPE	nd	[46]
QIAseq TMB panel	Qiagen	486	nd	Yes	SNVs, indels, and CNVs	Illumina NGS systems or Ion Torrent NGS systems	FFPE, plasma/serum, fresh or frozen tissue, cell lines	2–3 days	[47]
AVENIO ctDNA Targeted Kit	Roche	17	0.081	No	SNVs, indels, CNVs, and fusions	Illumina NextSeq 550	Plasma	5 days	[48]
Tempus xT v2	Tempus	596	nd	Yes	SNVs, indels, CNVs, genomic rearrangements, and MSI	Illumina HiSeq 4000	FFPE, frozen tissue, peripheral whole blood	9–14 days	[49]
Tempus xT v3	Tempus	648	3.6	No	SNVs, indels, CNVs, genomic rearrangements, MSI, and HRD	Illumina NovaSeq 6000	FFPE	9–14 days	[50]
Tempus xF Gene Panel	Tempus	105	nd	No	SNVs, indels, CNVs, and chromosomal rearrangements	Illumina NovaSeq 6000	Peripheral whole blood	nd	[51]
Oncomine Comprehensive Assay Plus	Thermo Fisher Scientific	>500	nd	Yes	SNVs, indels, structural rearrangements, CNVs, MSI, and HRD	Ion GeneStudio S5	FFPE	5 days	[24]
Oncomine Comprehensive Panel_v3 DNA	Thermo Fisher Scientific	161	0.39	No	Hotspots, CNVs, and fusions	Ion GeneStudio S5 or Genexus	FFPE	3 days	[52]
Oncomine Pan-Cancer Cell-Free Assay	Thermo Fisher Scientific	52	nd	No	SNVs, short indels, CNVs, and fusions	Ion GeneStudio S5	Peripheral whole blood	4 days	[53]
Oncomine Focus Assay DNA	Thermo Fisher Scientific	52	nd	No	SNVs, indels, CNVs, and fusions	Ion GeneStudio S5, S5 Plus or S5 Prime	FFPE	3 days	[54]
Oncomine Tumor Mutation Load Assay	Thermo Fisher Scientific	409	1.65	Yes	SNVs, indels, and CNVs	Ion GeneStudio S5	FFPE	3 days	[55]
